# Research on the influencing factors of subjective well-being of Chinese college students based on panel model

**DOI:** 10.3389/fpsyg.2024.1366765

**Published:** 2024-05-09

**Authors:** Ting Qin, Pingqiang Wei, Chengyi Zhu

**Affiliations:** ^1^School of Literature and Journalism, Xihua University, Chengdu, China; ^2^Xihua University Yibin Branch Management Committee, Yibin, China; ^3^School of Computer and Software Engineering, Xihua University, Chengdu, China

**Keywords:** college students, subjective well-being, panel model, confrontational interpretative structural model, CGSS, influencing factors

## Abstract

The subjective well-being of Chinese college students has always been a topic of concern. Subjective well-being is an overall evaluation of the quality of life according to the standards set by individuals, which is of great significance to the development of college students. Based on the data published in the past 5 years of China’s comprehensive social survey, this study uses panel model and adversarial explanatory structure model to analyze the influencing factors of subjective well-being of Chinese college students from five dimensions: social equity attitude, parental education, use of network, social interaction and physical health. The results show that social justice attitude, parents’ education, network use, social interaction and physical health have a positive impact on the subjective well-being of Chinese college students. Among them, the use of the network and the education of parents mainly affect the social justice attitude, social interaction attitude, physical health status, and ultimately affect the subjective well-being of college students. Based on the above conclusions, this study proposes strategies to improve the subjective well-being of college students, which has certain reference and guiding significance for educators and decision makers, and has reference significance for developing countries.

## 1 Introduction

Since China’s reform and opening up, with the social progress and the improvement of people’s living standards, the attention to subjective well-being is also increasing. Subjective well-being is a comprehensive concept, including subjective evaluation of individual life satisfaction, happiness and well-being (Ryan and Willits, 2007; Diener and Chan, 2011). In this context, many studies have been devoted to exploring the factors that affect subjective well-being.

Chinese college students are an important research object. They are one of the main forces of social development. Their subjective well-being is not only related to their own happiness, but also to the development and stability of family, society and country (Tay et al., 2014; Diener et al., 2018; Chi et al., 2019). Therefore, it is of great theoretical and practical significance to study the influencing factors of subjective well-being of Chinese college students, which is of reference significance to developing countries.

In the existing research, scholars mainly focus on the influence of emotional, psychological, personality and consciousness factors on the subjective well-being of college students (Wang et al., 2014; Sánchez-Álvarez et al., 2016; Liu et al., 2019). For example, trait mindfulness can significantly and negatively predict college students’ loneliness (Jin et al., 2020). Individual authenticity not only directly affects college students’ subjective well-being, but also affects college students’ subjective well-being through mindfulness and self-determination (Li et al., 2021). Dispositional optimism significantly affects subjective well-being, and perceived social support plays a mediating role in the relationship between dispositional optimism and subjective well-being (Gong et al., 2021; Zhang et al., 2022; Zhu et al., 2022). The intervention effect of dormitory culture and group counseling on subjective well-being of college students (Gao et al., 2016; Tang et al., 2016). Parents’ parenting style and emotional warmth affect college students’ subjective well-being (Zhou et al., 2014; He and Pei, 2015). Self-compassion and extroverted personality make college students have higher subjective well-being (Li et al., 2015; Ge et al., 2019; Margolis et al., 2020). These results show that positive emotional factors have a positive guiding effect on college students’ subjective well-being.

In addition, some studies have focused on the relationship between college students’ participation in various types of learning, social practice and subjective well-being (Renshaw, 2018; Zhang and Carciofo, 2021). For example, social connections and interactions affect college students’ subjective well-being (Lee et al., 2008). Students with high subjective well-being are more enthusiastic about college life than their peers with low subjective well-being (Antaramian, 2015; Zhang et al., 2017). Physical exercise enables college students to effectively cope with and adapt to setbacks and pressures in life, maintain a stable state of mind, and ultimately perceive more happiness (Chen and Yu, 2015; Zhou and Zhou, 2022). Voluntary service participation can directly and indirectly affect college students’ subjective well-being (Peng, 2022). These studies have shown that more participation in physical exercise, social practice, and various activities can help improve the subjective well-being of college students.

In addition, studies have shown that the use of social media has a certain impact on subjective well-being. For example, subjective well-being and self-control have a significant negative predictive effect on Internet addiction (Zhou and Zhou, 2017; Niu et al., 2018; Shi et al., 2018). Self-portrait editing has a positive feedback effect on the subjective well-being of female college students (Meng et al., 2017). College students’ We Chat use intensity has nothing to do with subjective well-being (Liu, 2018). The use of social media by emerging adults is the result of emotional regulation difficulties. These difficulties will have an impact on perceived stress and mental health, and ultimately affect the subjective well-being of college students (Rasmussen et al., 2020). These results show that the use of social media has a negative impact on college students’ subjective well-being.

In summary, the existing research mainly focuses on a certain influencing factor, which is neither comprehensive nor in-depth. This study not only comprehensively discusses the influence of social justice attitude, parents’ education, network use, social interaction and physical health on college students’ subjective well-being, but also innovatively uses panel model and adversarial interpretative structural model to study the influencing factors of Chinese college students’ subjective well-being. It is different from the past in terms of research methods, data sources, and selection of influencing factors. The aim is to make our research more comprehensive and systematic, so as to provide theoretical support and practical reference for improving the subjective well-being of college students, provide reference for educators and policy makers, and promote the sustainable development of education and human society.

## 2 Data sources and research methods

### 2.1 Data sources

In order to explore the influencing factors of subjective well-being of Chinese college students, this study uses the Chinese General Social Survey from 2013 to 2021 for research and analysis (as of October 16, 2023, we selected the latest data of the latest years that have been released free of charge, namely 2021, 2018, 2017, 2015, 2013). The China General Social Survey, started in 2003, is a continuous research project carried out by the China Survey and Data Center of Renmin University of China. It collects data at all levels in China comprehensively and systematically. It has the characteristics of continuity, comprehensiveness and authority. In the following, we refer to the China General Social Survey as CGSS. For example, the 2013 China Comprehensive Social Survey is referred to as CGSS (2013), the 2015 China Comprehensive Social Survey is referred to as CGSS (2015), the 2017 China Comprehensive Social Survey is referred to as CGSS (2017), the 2018 China Comprehensive Social Survey is referred to as CGSS (2018), and the 2021 China Comprehensive Social Survey is referred to as CGSS (2021). There are 8,148 original samples of CGSS (2021), 12,787 original samples of CGSS (2018), 12,582 original samples of CGSS (2017), 10,968 original samples of CGSS (2015) and 11,438 original samples of CGSS (2013). According to the research needs, the original sample data were sorted, eliminated and cleaned, and finally a total of 1,524 research sample data were obtained.

In view of this research question, six variables are set up for discussion. Select “social justice attitude”, “parents” education”, “use of the Internet”, “social situation”, “physical health” and “subjective well-being” as variables:

#### 2.1.1 Social equity attitude

This study selects the question “A35” in CGSS (2021). In general, do you think that today’s social equity is unfair? This problem is selected as the research variable of this study. The “completely unfair” is assigned to 1; “Comparative unfairness” is assigned to 2; assign “not fair but can not say unfair” to 3; the “comparative fairness” is assigned to 4; the “complete fairness” is assigned to 5. The higher the value, the higher the degree of fairness.

#### 2.1.2 Parental education

This study selected the question “A89b” in CGSS (2021). What is your father’s highest education level? A90b. What is your mother’s highest education level? These two questions are selected as the research variables of this study. Assign “no education” to 1; the “literacy class” was assigned to 2; the value of “primary school” is assigned to 3; assign “junior high school” to 4; to assign “vocational high school” to 5; the “ordinary high school” is assigned to 6; the value of “technical secondary school” is assigned to 7; the “technical school” is assigned to 8; the “college (adult higher education)” is assigned to 9; the “college (formal higher education)” is assigned to 10; the “undergraduate (adult higher education)” is assigned to 11; the “university undergraduate (formal higher education)” is assigned to 12; “Graduate students and above” was assigned to 13. The higher the value, the higher the education level.

#### 2.1.3 Use of the internet

This study selects the question “A30.12” in CGSS (2021). In the past 1 year, have you often engaged in online activities in your free time? This problem is selected as the research variable of this study. Assign “never” to 1; assign “1 year several times or less” to 2; assign “several times a month” to 3; the value of “several times a week” is assigned to 4; assign “everyday” to 5. The higher the value, the more frequent the Internet.

#### 2.1.4 Social situation

This study selected the question “A31b” in CGSS (2021). What is the frequency of social entertainment activities with other friends (such as visiting each other, watching TV together, eating, playing cards, etc.)? This problem is selected as the research variable of this study. Assign “Never” to 1; assign “once a year or less” to 2; assign “several times a year” to 3; assign “about once a month” to 4; assign “several times a month” to 5; assign “1 to 2 times a week” to 6; assign “almost every day” to 7. The higher the value, the higher the degree of social interaction, the better the social situation.

#### 2.1.5 Physical health status

This study selected the question “A15” in CGSS (2021). What do you think your current physical health status is? This problem is selected as the research variable of this study. Assign “very unhealthy” to 1; to assign the value of “relatively unhealthy” to 2; “General” is assigned to 3; the “comparative health” is assigned to 4; assign “very healthy” to 5. The higher the value, the higher the degree of health.

#### 2.1.6 Subjective well-being

This study selected the question “A36” from CGSS (2021). In general, do you think your life is happy? This problem is selected as the research variable of this study. Assign “very unhappy” to 1; “Comparative unhappiness” was assigned to 2; assign “not happy” to 3; the “comparative happiness” is assigned to 4; assign “very happy” to 5. The higher the value, the stronger the degree of happiness.

### 2.2 Descriptive statistics of data

**Figure 1:**
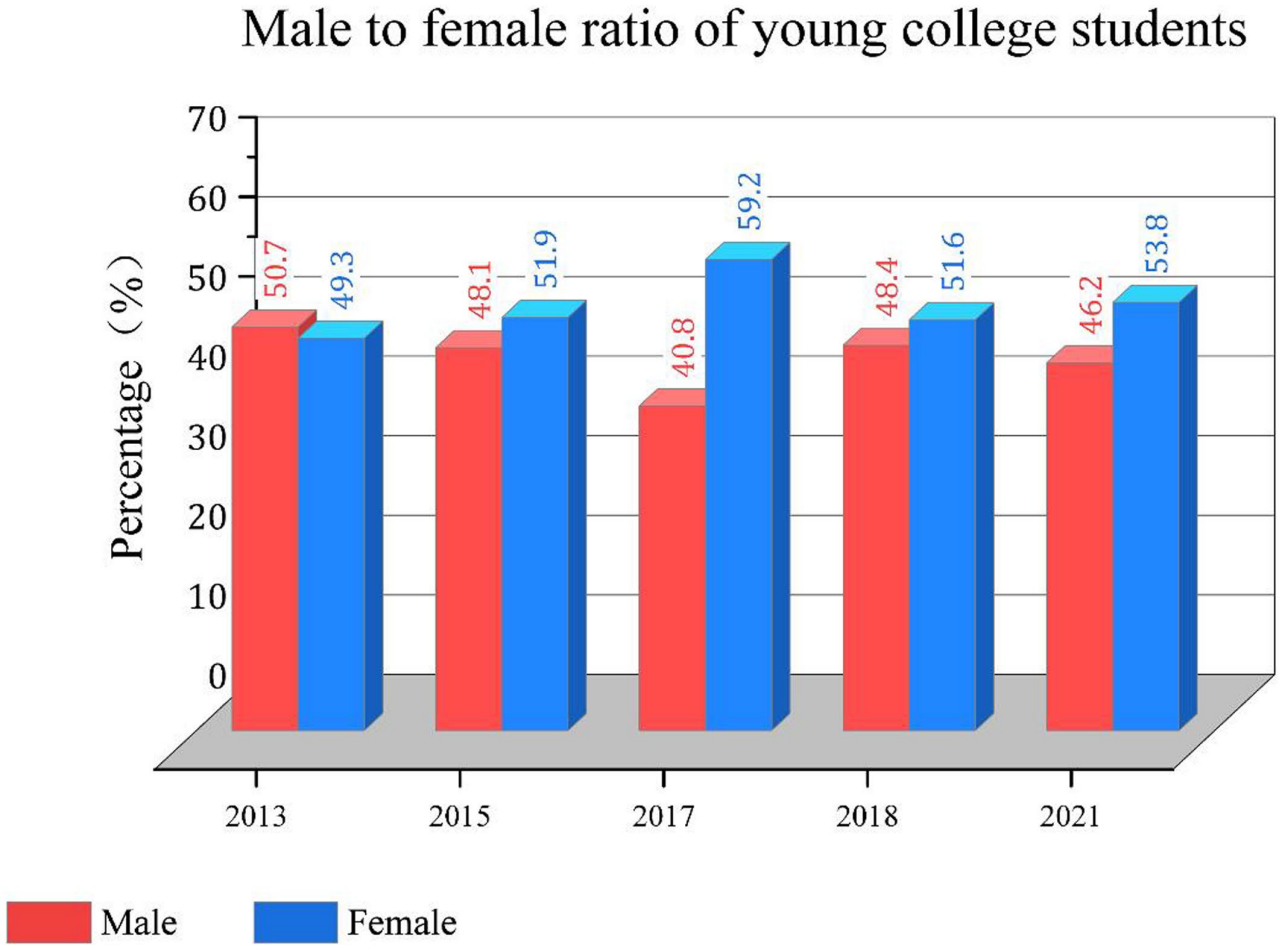
Male to female ratio of college students.

**Figure 2:**
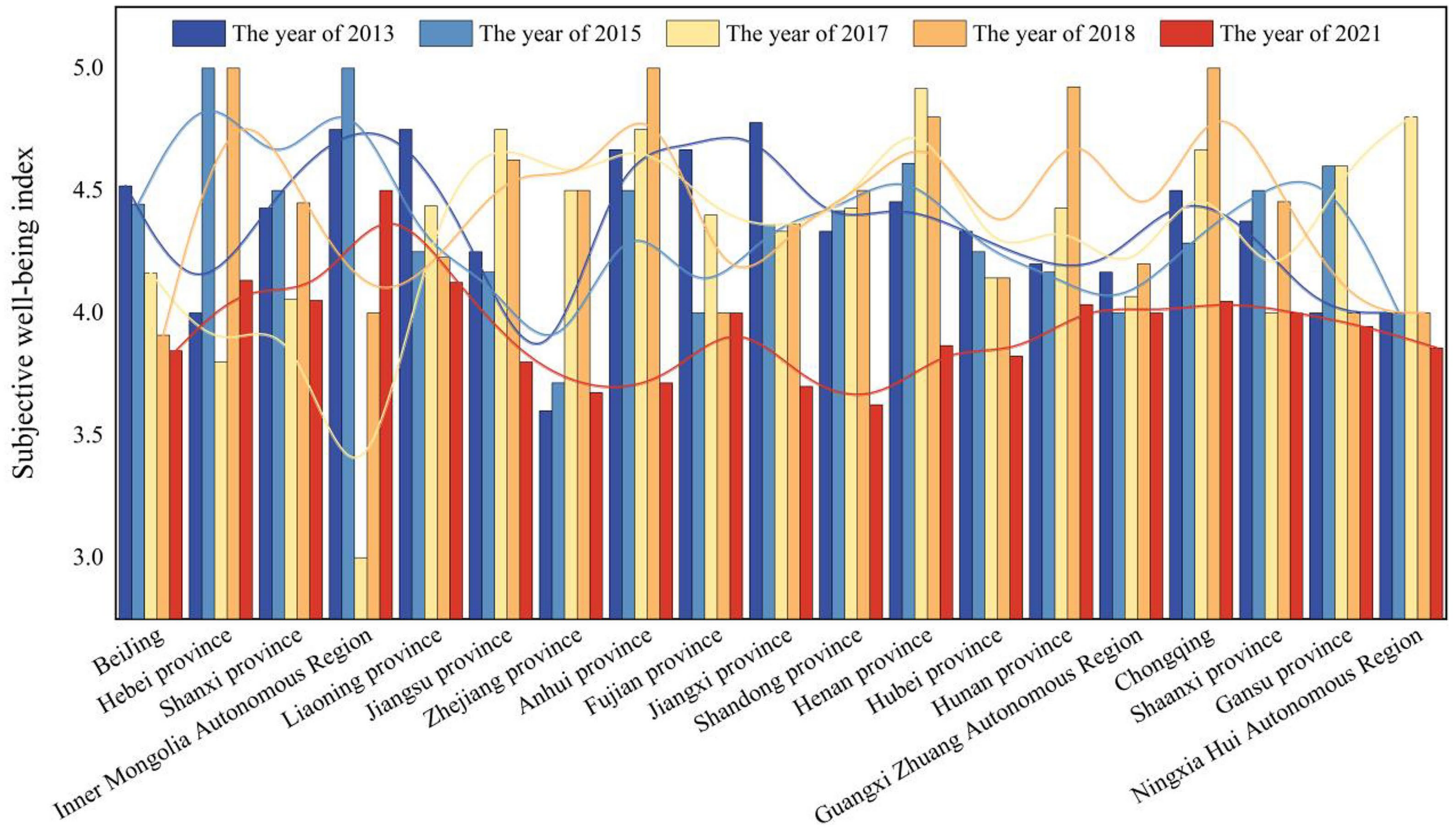
The change of subjective well-being of college students in various provinces and cities.

For the sample data of this study, the proportion of men and women in this study is basically stalemate, and the data distribution is universal. The results are shown in Figure 1. Using the data of 2013, 2015, 2017, 2018 and 2021, the trend of subjective well-being of college students in these 19 provinces and cities is calculated. The specific content is shown in Figure 2.

### 2.3 Method

This study uses panel regression model to discuss. Panel regression is an econometric model based on panel data to analyze the relationship between variables. The panel data selected in this study are the data of college students in 19 provinces and cities in China in 2021, 2018, 2017, 2015 and 2013, respectively. The data includes data on six variables: social equity attitudes, parental education, use of the Internet, social networking, physical health, and subjective well-being. Panel models are usually divided into three categories, namely mixed model, fixed effect model and random effect model. The formulas of the mixed model and the fixed effect model are expressed as follows:

Mixed model:


(1) $$y_{\mathrm{it}}=\alpha +x_{\mathrm{it}}\beta +\mu_{\mathrm{it}},i=1,2\ldots N;j=1,2,\ldots T$$

Fixed effect model:


(2) $$y_{\mathrm{it}}=\lambda_{i}+\overset{K}{\underset{k=2}{\sum }}\beta_{k}x_{\mathrm{kit}}+u_{\mathrm{it}}$$

(3) $$y_{\mathrm{it}}=\gamma_{i}+\overset{K}{\underset{k=2}{\sum }}\beta_{k}x_{\mathrm{kit}}+u_{\mathrm{it}}$$

(4) $$y_{\mathrm{it}}=\lambda_{i}+\gamma_{i}+\overset{K}{\underset{k=2}{\sum }}\beta_{k}x_{\mathrm{kit}}+u_{\mathrm{it}}$$

In the panel model, the individual items of this study are 19 provinces and cities (Beijing, Hebei Province, Shanxi Province, Inner Mongolia Autonomous Region, Liaoning Province, Jiangsu Province, Zhejiang Province, Anhui Province, Fujian Province, Jiangxi Province, Shandong Province, Henan Province, Hubei Province, Hunan Province, Guangxi Zhuang Autonomous Region, Chongqing City, Shaanxi Province, Gansu Province, Ningxia Hui Autonomous Region); the time items are 2021, 2018, 2017, 2015 and 2013; the dependent variable is the subjective well-being of college students; the independent variables are social equity attitudes, parental education, use of the Internet, social networking, and physical health.

### 2.4 Research hypothesis

This study takes Chinese college students as the research object to explore the influencing factors of subjective well-being of Chinese college students. Based on CGSS data, this study uses panel model to explore the relationship between subjective well-being of Chinese college students and social equity attitude, network use, social situation, physical health status and parents’ education level, and puts forward the following five hypotheses:

Whether the individual cognitive society is fair or not is the main judgment standard of social equity attitude. Among them, whether income is equal (Su and Zhang, 2021; Zhang and Zhang, 2023), participation in pension insurance and medical insurance (Wang and Chen, 2022), physical health status (Wang, 2019), and pro-environmental behavior (Yang and Hu, 2018) affect the cognitive attitude towards social justice and directly affect the degree of subjective well-being. Social justice has a direct contribution to subjective well-being (Wang et al., 2023), indirectly affects residents’ life satisfaction (Feng et al., 2023), and affects individuals’ enthusiasm for political participation (Zhang and Ge, 2023). Based on this, this study proposes *Hypothesis 1*: Good social justice attitude will positively affect the subjective well-being of Chinese college students.

The higher the education level of parents, the more time and money they are willing to invest in their children, so that children and families can achieve healthy and sustainable development. Parents’ education level can significantly improve their children’s cognitive and non-cognitive abilities (Chen et al., 2024), and has a significant positive income effect on their offspring’s income (Fan and Zhao, 2023). The growth process of children, especially the positive impact and the socioeconomic status in adulthood are affected to varying degrees, and there is a positive correlation with subjective well-being (Sutin et al., 2018). Based on this, this study proposes *Hypothesis 2*: Good parental education has a positive impact on the subjective well-being of Chinese college students.

The use of the network is a double-edged sword, which can greatly meet the needs of people’s life. If you indulge in the network, you will hurt your body and mind. Different types of Internet use have different effects on subjective well-being (Donoso et al., 2021; Zhang, 2023). In particular, social network use has a direct impact on subjective well-being (Cao, 2021). The information acquisition behavior in primary school students’ Internet use behavior is significantly positively correlated with subjective well-being (Song and Yu, 2020), but Internet use is significantly negatively correlated with the life satisfaction of the elderly (Yang et al., 2021). Based on this, this study proposes *Hypothesis 3*: The moderate use of the network helps to improve the subjective well-being of Chinese college students.

Social activities are a significant sign that human beings are different from other animals. With the development of Internet of Things technology, more and more people are willing to communicate on the Internet and actively use online social networking, which is conducive to the improvement of subjective well-being (Verduyn et al., 2017), in order to meet their own needs (Zhou and Wang, 2023). In offline life, we also inevitably need to communicate. Better social relations (Diener et al., 2018) are for us to better socialize. Positive mentality and emotion can improve the level of subjective well-being (Joshanloo et al., 2018). Based on this, this study proposes *Hypothesis 4*: Positive social interaction is related to the subjective well-being of Chinese college students.

There is a positive relationship between physical exercise and physical health. The study found that physical exercise has little effect on subjective well-being (Buecker et al., 2021). Everyone attaches great importance to their physical health. Whether the body is healthy or not directly affects subjective well-being (Mo, 2023), but health values are not related to subjective well-being (Dobewall et al., 2018). Social integration, life satisfaction, environmental pollution, infrastructure, etc., all affect the individual’s physical health and subjective well-being (Xu et al., 2023). Based on this, this study proposes *Hypothesis 5*: There is a positive relationship between good physical health and subjective well-being of Chinese college students.

## 3 Empirical analysis and results

### 3.1 Panel data analysis

Based on the data of 19 provinces and cities in 2013, 2015, 2017, 2018 and 2021, this study establishes a panel model to explore whether the five factors of social equity attitude, parents’ education, use of network, social situation and physical health status have an impact on the subjective well-being of Chinese college students. The model data sample is summarized, as shown in Table 1.

**Table 1:** Sample missing summary.

Item	Number of samples	Proportion
Valid samples	95	100.0%
Incomplete samples	0	0.0%
Grand total	92	100.0%

As shown in Table 1, “valid sample” refers to the total number of samples in which all analysis items have data, and “missing sample” refers to the total number of samples missing in any analysis item.

The panel regression model usually involves three models, namely the mixed POOL model, the fixed effect FE model and the random effect RE model. For the selection of the model, this study will synthesize the *F* test, the Breusch-Pagan test, and the Hausman test. The results of these three tests are used to select the most suitable model. The test results are shown in Table 2.

**Table 2:** Summary of test results.

*N* = 95			
Inspection type	Check purpose	Monitor value	Inspection conclusion
*F* test	FE model, POOL model Compare options	*F* (18,71) = 0.623 *p* = 0.870	POOL model
BP test	FE model, POOL model Compare options	$\chi^{2}$ (1) = 1.537 *p* = 0.107	POOL model
Hausman test	FE model, RE model Compare options	$\chi^{2}($ 4) = −0.476 *p* = 1.000	RE model

In this study, the degree of social equity attitude, the degree of Internet access, the degree of social interaction, the degree of physical health, the degree of parental education as explanatory variables, and the degree of subjective well-being as explanatory variables were used to construct the panel model for Chinese college students, and the robust standard error method was used for modeling. It can be seen from Table 2 that, firstly, the F test does not show a significant *F*(18,71) = 0.623, *p* = 0.870 > 0.05, which means that the POOL model is better than the FE model. Secondly, the Breusch-Pagan test did not show significant $\chi^{2}$ (1) = 1.537, *p* = 0.107 > 0.05, which means that the POOL model is better than the RE model. Finally, the Hausman test did not show a significant $\chi^{2}$ (4) = −0.476, *p* = 1.000 > 0.05, which means that the RE model is better than the FE model. Based on the above analysis, this study finally chose the POOL model as the optimal model.

In this study, the POOL model was selected for analysis. If a certain item showed a significant (*p* < 0.05), it means that the item has an impact on the dependent variable (explained variable), and vice versa. The relationship is not affected. The results of the panel regression model are shown in Tables 3, 4.

**Table 3:** Panel model results (POOL model).

Dependent variable: the degree of subjective well-being			
Item	Coef.	Std. Err	*t*
Intercept	4.096	0.957	4.280
Social justice attitude	0.920	0.112	9.757
Use internet	0.222	0.131	2.503
Social situation	0.287	0.122	2.632
Health status	0.463	0.071	3.741
Parents’ education	0.523	0.038	4.976

**Table 4:** Panel model results (POOL model).

Dependent variable: the degree of subjective well-being			
Item	*p*	95% CI	
Intercept	0.000^**^	2.220 ~ 5.971	^**^p = 0.000 *R*^2^ = 0.951 *R*^2^ (within) = 0.982 ^*^p < 0.05 ^**^p < 0.01
Social justice attitude	0.000^**^	−0.165 ~ 0.273	
Use internet	0.001^**^	−0.317 ~ 0.195	
Social situation	0.000^**^	−0.227 ~ 0.250	
Health status	0.009^**^	−0.057 ~ 0.220	
Parents’ education	0.000^**^	−0.092 ~ 0.058	

This study uses the POOL model as the final result. As shown in Tables 3, 4, for the degree of social fairness attitude, the result shows significance (*t* = 9.757, *p* = 0.000 < 0.01), and the regression coefficient value is 0.920 > 0, indicating that the degree of social fairness attitude has a significant positive impact on the subjective well-being of Chinese college students. For the use of the Internet, the result shows significance (*t* = 2.503, *p* = 0.001 < 0.01), and the regression coefficient value is 0.222 > 0, indicating that the use of the Internet has a significant positive impact on the subjective well-being of Chinese college students. For social conditions, the result shows significance (*t* = 2.632, *p* = 0.000 < 0.01), and the regression coefficient value is 0.287 > 0, indicating that social conditions have a significant positive impact on the subjective well-being of Chinese college students. For physical health status, the result shows significance (*t* = 3.741, *p* = 0.009 < 0.01), and the regression coefficient value is 0.463 > 0, indicating that physical health status has a significant positive impact on the subjective well-being of Chinese college students. For parents’ education, the result shows significance (*t* = 4.976, *p* = 0.000 < 0.01), and the regression coefficient value is 0.523 > 0, indicating that parents’ education has a significant positive impact on the subjective well-being of Chinese college students.

To sum up, it can be proved that the hypothesis of this study is true. The subjective well-being of Chinese college students is influenced by five dimensions: social justice attitude, network usage, social situation, physical health and parents’ education level.

### 3.2 Analysis of influencing factors

After the previous article has proved that Chinese college students’ subjective well-being and social justice attitude, network usage, social situation, physical health and parents’ education level have an impact on the premise. In order to further explore the relationship between the factors affecting the subjective well-being of Chinese college students, this study further uses the resonance confrontation explanatory structure model (RAISM) for analysis.

**Figure 3:**
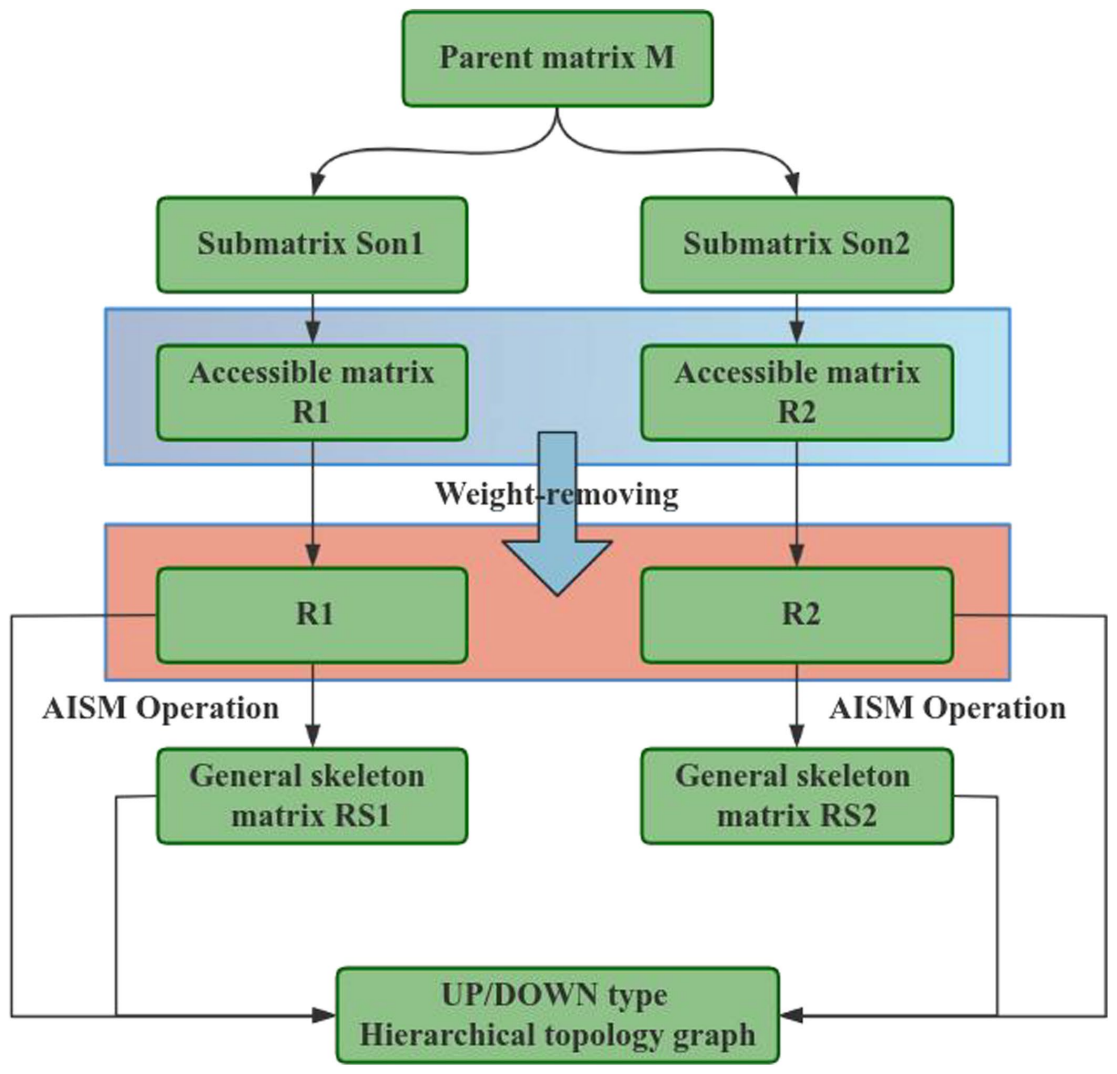
Flow chart of calculation steps.

Resonant Antagonism Interpretative Structure Model (RAISM) is an improved model that introduces uncertainty relationship on the basis of Antagonism Interpretative Structure Model (AISM). The RAISM method contains a number of concepts, such as parent matrix, resonance body, resonance structure, sub-matrix, uncertainty relation and so on. Among them, the parent matrix represents the data set to be explained, the resonance body refers to the resonance relationship between these data, and the uncertainty relationship refers to the possibility that there may be a reachable relationship or no reachable relationship between the two influencing factors. The flow chart of the model calculation steps is shown in Figure 3.

This model selects social equity attitude, parents’ education level, network use, social situation, physical health, college students’ subjective well-being as the factors of model analysis. The original matrix of this model is shown in Table 5. The original matrix represents the initial relationship between the factors in this study.

**Table 5:** Matrix *M_matrix.*

M_6 × 6_	Social justice attitude	Parents’ education level	Use internet	Social intercourse	Physical health status	The subjective well-being
Social justice attitude	0	0	0	0	0	1
Parents’ education level	1	0	0	1	0	1
Use internet	0	0	0	0	1	1
Social intercourse	0	0	0	0	0	1
Physical health status	1	0	0	※	0	1
The subjective well-being	0	0	0	0	0	0

※Indicates uncertain relationship.

Table 5 is shown as the matrix *M_matrix* containing the uncertainty relation. There is an uncertainty relation in the matrix, which corresponds to the first power of 2, that is, two matrices. Therefore, there are two sub-matrices to determine the substructure. The results of the two sub-matrices are shown in Tables 6, 7.

**Table 6:** Submatrix Son1.

M_6 × 6_	Social justice attitude	Parents’ education level	Use internet	Social intercourse	Physical health status	The subjective well-being
Social justice attitude	1	0	0	0	0	1
Parents’ education level	1	1	0	1	0	1
Use internet	0	0	1	0	1	1
Social intercourse	0	0	0	1	0	1
Physical health status	1	0	0	0	1	1
The subjective well-being	0	0	0	0	0	1

**Table 7:** Submatrix Son2.

M_6 × 6_	Social justice attitude	Parents’ education level	Use internet	Social intercourse	Physical health status	The subjective well-being
Social justice attitude	1	0	0	0	0	1
Parents’ education level	1	1	0	1	0	1
Use internet	0	0	1	0	1	1
Social intercourse	0	0	0	1	0	1
Physical health status	1	0	0	1	1	1
The subjective well-being	0	0	0	0	0	1

According to the results of the sub-matrices in Tables 6, 7, after deduplication, the reachable matrix R is obtained, and the result of the reachable matrix R is shown in Table 8.

**Table 8:** Reachable matrix R.

M_6 × 6_	Social justice attitude	Parents’ education level	Use internet	Social intercourse	Physical health status	The subjective well-being
Social justice attitude	1	0	0	0	0	1
Parents’ education level	1	1	0	1	0	1
Use internet	1	0	1	0	1	1
Social intercourse	0	0	0	1	0	1
Physical health status	1	0	0	0	1	1
The subjective well-being	0	0	0	0	0	1

Then use the reachable matrix R in Table 8 to calculate the reduced point reachable matrix R’ and the skeleton matrix S’. The calculation formula is as follows:


(5) $$S’=R’-{\left(R’-I\right)}^{2}-I$$

As shown in Table 9, it represents the result that the contraction point can reach the matrix R’ calculated by Formula (5).

**Table 9:** Shrinking point reachable matrix R’.

M_6 × 6_	Social justice attitude	Parents’ education level	Use internet	Social intercourse	Physical health status	The subjective well-being
Social justice attitude	1	0	0	0	0	1
Parents’ education level	1	1	0	1	0	1
Use internet	1	0	1	0	1	1
Social intercourse	0	0	0	1	0	1
Physical health status	1	0	0	0	1	1
The subjective well-being	0	0	0	0	0	1

As shown in Table 10, it represents the result of the skeleton matrix S’ calculated by Formula (5).

**Table 10:** Skeleton matrix S’.

M_6 × 6_	Social justice attitude	Parents’ education level	Use internet	Social intercourse	Physical health status	The subjective well-being
Social justice attitude	1	0	0	0	0	1
Parents’ education level	1	1	0	1	0	0
Use internet	0	0	1	0	1	0
Social intercourse	0	0	0	1	0	1
Physical health status	1	0	0	0	1	0
The subjective well-being	0	0	0	0	0	1

Then according to the model, according to the two levels of “result priority” and “cause priority” extraction rules, the extraction of hierarchical division is carried out, and the extraction process is shown in Table 11.

**Table 11:** Level extraction process.

Result first – UP type extraction			Reason first – DOWN type extraction		
	R_e_	T_e_		Q_e_	T_e_
A1	A1, A6	A1	A1	A1, A2, A5	A1
A2	A1, A2, A4	A2	A2	A2	A2
A3	A3, A5	A3	A3	A3	A3
A4	A4, A6	A4	A4	A2, A4	A4
A5	A1, A5	A5	A5	A3, A5	A5
A6	A6	A6	A6	A1, A4, A6	A6
**Extract the subjective well-being**			**Extract the parents’ education level, use internet**		
	R_e_	T_e_		Q_e_	T_e_
A1	A1	A1	A1	A1, A5	A1
A2	A1, A2, A4	A2	A4	A4	A4
A3	A3, A5	A3	A5	A5	A5
A4	A4	A4	A6	A1, A4, A6	A6
A5	A1, A5	A5			
**Extract the social justice attitude, social activities**			**Extract the social activities, health status**		
	R_e_	T_e_		Qe	T_e_
A2	A2	A2	A1	A1	A1
A3	A3, A5	A3	A6	A1, A6	A6
A5	A5	A5			
**Extract the parents’ education level, health status**			**Extract the social justice attitude**		
	R_e_	T_e_		Q_e_	T_e_
A3	A3	A3	A6	A6	A6

As shown in Table 11, it shows the extraction results of hierarchical division according to the two hierarchical extraction rules of “result priority” and “cause priority”.

As shown in Table 12, the results of the model extracted according to the RAISM model are as follows: UP type ≺ {(young college students’ subjective well-being)} ≺ {(social justice attitude, social situation)} ≺ {(parents’ education level, physical health)} ≺ {(network usage)}; DOWN type ≺ {(subjective well-being of young college students)} ≺ {(social equity attitude)} ≺ {(social situation, physical health)} ≺ {(parents’ education level, network usage)}.

**Table 12:** Extract the result.

Hierarchy	Results priority – UP type	Cause priority – DOWN type
Layer 0	College students’ subjective well being	College students’ subjective well being
Layer 1	Social justice attitude, social situation	Social justice attitude
Layer 2	Parents’ education level and physical health	Social situation, physical health
Layer 3	Internet use behaviors	Parents’ education level and network usage

**Figure 4:**
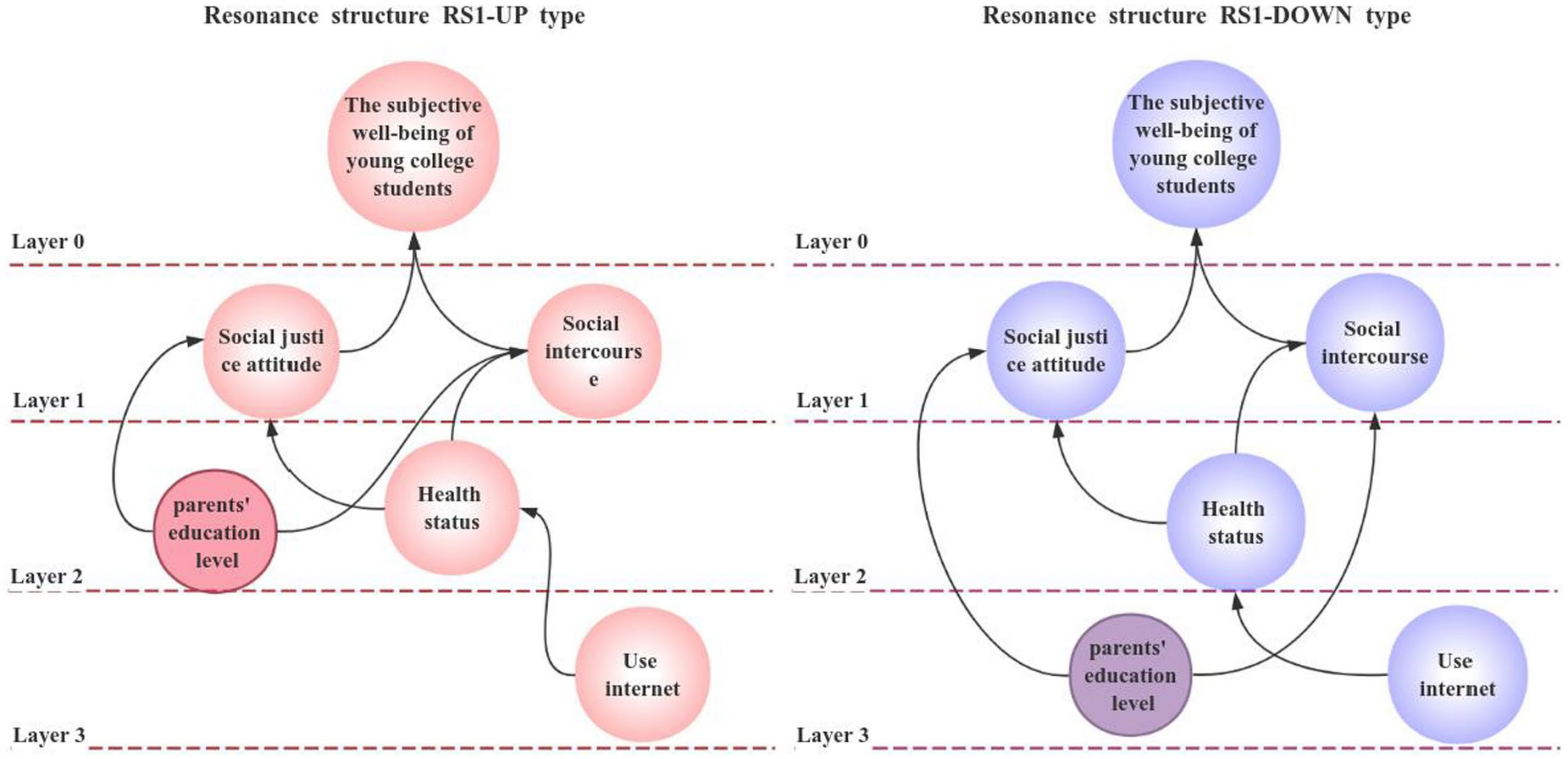
Resonance structure RS1 against hierarchical topology diagram.

**Figure 5:**
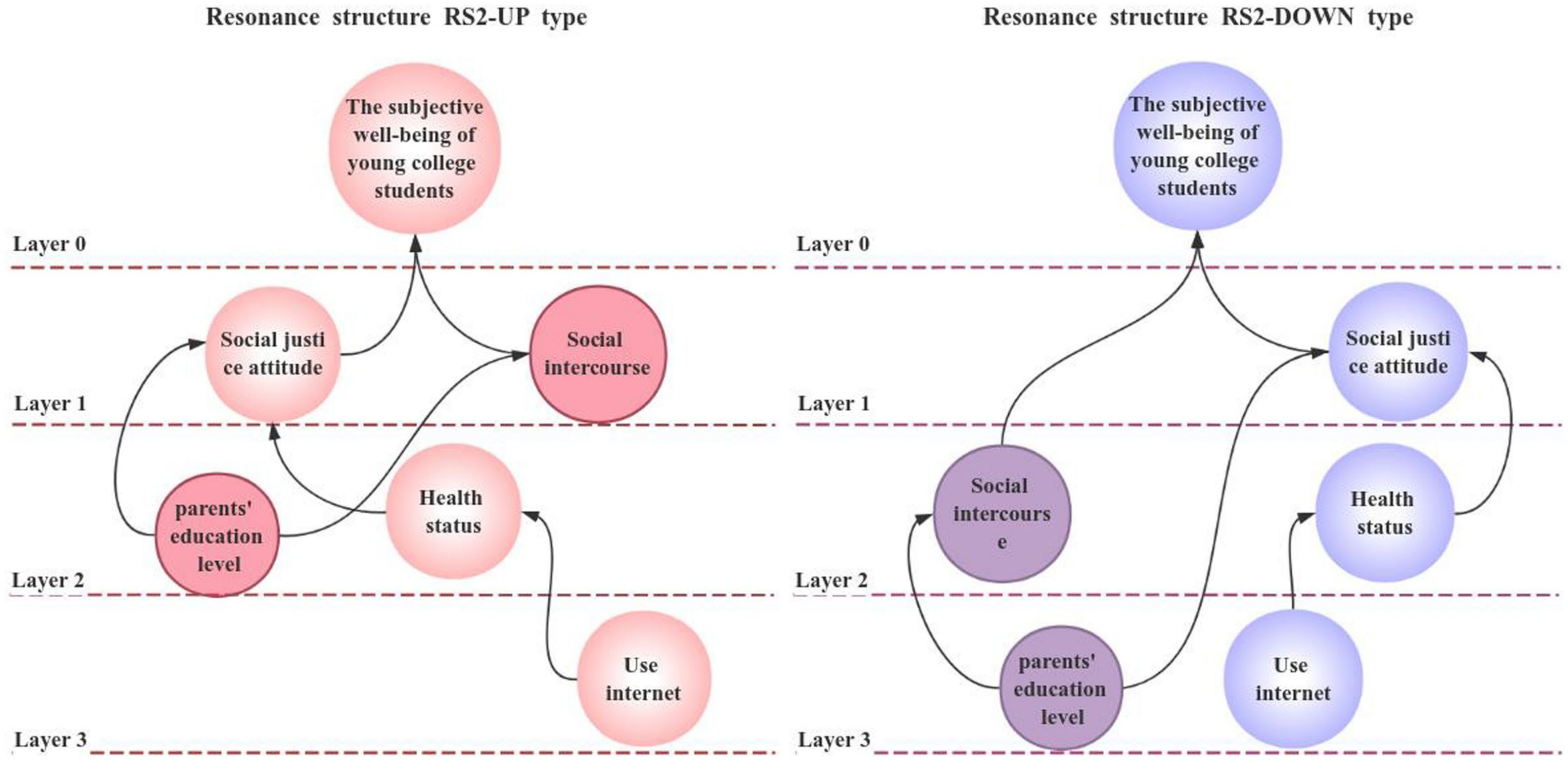
Resonant structure RS2 against hierarchical topology.

Finally, according to the results of the model, the confrontation level topology of all resonance structures is drawn, as shown in Figures 4, 5.

From Figures 4, 5, we can see that under the study of this model, the subjective well-being of Chinese college students has an influence relationship with social justice attitude, network usage, social situation, physical health status and parents’ education level. Specifically, the use of the Internet will affect the physical health of Chinese college students, physical health will affect the attitude of college students to social justice. At the same time, the education level of parents will also affect the degree of college students’ attitude towards social equity and social situation. Finally, the degree of subjective well-being of college students is affected by social equity attitude factors and social situation factors.

## 4 Discussion

Through the analysis of panel model and resonance confrontation explanatory structure model, this study explores the influence of subjective well-being and social equity attitude, network use, social situation, physical health status and parents’ education level of Chinese college students.

First, a good social justice attitude will have a positive impact on the subjective well-being of Chinese college students. This is because in a social environment that is considered fair and just, individuals are more likely to feel justice and have a positive impact on personal development, thereby improving subjective well-being (Sun and Jin, 2016). It should be noted that the perception of social justice is subjective (Jiang et al., 2017), and different individuals may have different definitions and feelings of justice. Some college students may have different views on social equity, which may lead to different degrees of impact on happiness.

Secondly, parents’ good education has a positive impact on college students’ subjective well-being. This is because well-educated parents are more able to provide better family education and support, which has a positive impact on the growth and development of their children (Sun and Li, 2015). However, parents’ education level is not the only influencing factor, and family economic status, family relationships, etc., may also have an impact on subjective well-being (Lampropoulou, 2018). Sometimes, too high expectations may also cause children to feel more pressure and reduce their happiness.

Third, the moderate use of the network helps to improve the subjective well-being of college students. This is because the rational use of the network helps to expand the social circle, obtain information, and improve the individual’s sense of social participation, thereby enhancing happiness (Shen et al., 2014). However, network usage may also cause some negative effects. For example, addiction to the Internet, social media anxiety and other issues (Wu, 2023; Zhang et al., 2023), which may have a negative impact on subjective well-being. Therefore, it is necessary to pay attention to the reasonable guidance and management of network use.

Fourth, positive social interaction is related to the subjective well-being of Chinese college students. This suggests that social situations contribute to the establishment of good interpersonal relationships and improve the sense of social integration, thus contributing to the improvement of subjective well-being (Ye et al., 2018). However, excessive social interaction may also lead to stress and fatigue (Ye et al., 2021; Valkenburg, 2022), especially at a faster pace of life in college. Therefore, there is a need to balance the frequency and intensity of social situations to ensure their positive impact.

Fifth, there is a positive relationship between good physical health and subjective well-being of college students. Physical health is the basis of well-being, which helps to improve individual’s life satisfaction and well-being (Sun et al., 2023). It should be noted that physical health may be affected by some unpredictable factors, such as disease, accidental injury, etc., (Petrides et al., 2016; Yang and Wang, 2023), which may have a negative impact on subjective well-being. Therefore, health care and preventive measures are still crucial.

There are two shortcomings in this study. First, we study the influencing factors of subjective well-being of Chinese college students. Whether it is universal for other non-college students and other countries needs further verification. Second, our data come from 2021 and before. With the update of time, place and group samples, whether the influencing factors will change is also a question worth considering.

In summary, the results of this study reveal the complex effects of multiple dimensions on the subjective well-being of Chinese college students, which helps to deepen the understanding of the formation mechanism of well-being and provides a reference for the formulation of well-being promotion policies. However, it is necessary to comprehensively consider multiple factors in the formulation of specific policies to improve the subjective well-being of college students more comprehensively and effectively.

## 5 Conclusion

Social justice attitude, parents’ education, use of the Internet, social interaction and physical health have a positive impact on the subjective well-being of Chinese college students. Specifically, the use of the Internet will affect the physical health status of Chinese college students, and the physical health status will affect the social equity attitude of college students. Parents’ education level will also affect college students’ attitude towards social equity and social situation. Social equity attitude factors and social situation factors affect the degree of subjective well-being of college students.

Based on this, we advocate the implementation of a wide range of education programs (Chen, 2016) aimed at raising public awareness of social equity. The education plan should include the concepts of fairness consciousness, equal opportunity and fair distribution, so as to cultivate college students’ sense of identity and behavior participation. Develop family-oriented educational support programs, provide parents with educational resources and guidance, emphasize the importance of family education to children’s subjective well-being, and encourage parents to pay attention to educational investment and family support in cultivating children (Jebb et al., 2018). To formulate guidelines for the healthy use of the Internet, to convey the importance of appropriate use of the Internet to college students, and to encourage the purposeful and restrained use of the Internet to protect college students from the negative effects of the Internet (Lei et al., 2020; Ye et al., 2023), to enhance their subjective well-being. Promote the diversity and inclusiveness of social situations, encourage college students to participate in associations, volunteer services and team activities, establish more social platforms, enhance college students’ social networks, and improve their sense of social integration. Strengthen college students’ physical health education and promote healthy lifestyles, including nutritional diet, regular exercise and mental health awareness. At the same time, implement preventive health strategies to reduce the negative impact of health problems on subjective well-being. Provide more opportunities for social participation, encourage college students to participate in public welfare activities, social volunteer services and civil society organizations, cultivate their sense of social responsibility and participation, and promote the improvement of subjective well-being.

## Data availability statement

The original contributions presented in the study are included in the article/supplementary material, further inquiries can be directed to the corresponding author.

## Ethics statement

The data used in this study are from the “Chinese General Social Survey” project hosted by the China Survey and Data Center of Renmin University of China. The author thanks this institution and its personnel for providing data assistance, and the content of this study is the author’s own responsibility.

## Author contributions

TQ: Conceptualization, Methodology, Writing – original draft. PW: Data curation, Resources, Supervision, Validation, Writing – review & editing. CZ: Formal analysis, Software, Writing – review & editing.
